# Ethnobotany of religious and supernatural beliefs of the Mising tribes of Assam with special reference to the 'Dobur Uie'

**DOI:** 10.1186/1746-4269-7-16

**Published:** 2011-06-02

**Authors:** Uma Kanta Sharma, Shyamanta Pegu

**Affiliations:** 1 Department of Botany, Dhemaji College, Dhemaji 787 057, Assam, India

## Abstract

Assam is very rich in plant biodiversity as well as in ethnic diversity and has a great traditional knowledge base in plant resources. It is inhabited by the largest number of tribes and they lead an intricate life totally dependent on forest plants. The Mising is the major section and second largest tribal community of Assam and have a rich tradition of religion and culture. Their religious practices and beliefs are based on supernaturalism. A study of the plants related to magico religious beliefs in Dobur Uie of Mising is carried out. The results revealed the use of 30 plants belonging to 23 families. All plant species are used both in religious purpose as well as in the treatment of different ailments. Details of the uses of plants and conservational practices employed in Dobur Uie are provided. Our findings on the use of plants in Dobur Uie ritual reflect that some plants are facing problems for survival and they need urgent conservation before their elimination. Because this elimination may threat the rich tradition of Mising culture. Most of the plants that are domesticated for different rituals are almost same in all Mising populated areas.

## Background

Plants are used in many ways including worshipping gods and goddesses for the protection and betterment of human life. In every human society worship is performed with traditional rituals for well-being. Many tribal communities preserve this tradition through folklore and worship their deities right from the occasion of birth to mourning death. They perform specific worship with pressie offerings.

In India various gods and goddesses are worshipped in different religions throughout the country. Various plant parts like bark, twigs, leaves flowers, fruits and seeds are offered to gods. There are many plants grown near the different religious institutions are regarded as sacred plants by different ethnic groups of the country. They preserve the plants by all means which are used in different rituals. At a time when ecological degradation and deforestation have been taking place at an alarming rate throughout the globe, in India thousands of pockets of natural vegetation scattered throughout the country are preserved almost in pristine condition [1-5]. Almost all the religious communities and tribal societies consider some plants as holy in origin and essential in religious functions.

Tribal folklore is rich in magico-religious beliefs and taboos. They believe that some gods and deities reside on the trees in the forest. If they do not show mark respect to them their full clan will be destroyed. So they preserve the plants which they regard sacred for social, cultural and religious purposes. Their taboos, festivals, rituals and other cultural aspects are closely associated with the surrounding vegetation preserved on religious ground. The fear of getting attacked by the forest spirits or getting cursed by the deities eventually makes the local communities to resort to worship the spirits and making sacrifices and offerings to pacify them. Although the taboos, self imposed restricted and extra care exhibited by the community have significantly contributed in preserving the religious plants intact and in good shape thereby conserving a whole range of biodiversity that is housed in it. There is an inextricable link between indigenous and biological diversity. All over the world the indigenous people have protected the biodiversity with which they have symbiotic relationship [6]. It has been an undeniable fact that the knowledge of indigenous people is invaluable in the present day context of biological diversity conservation and its sustainable utilization [7,8]. The use of plants in different religious practices is possibly the earliest and most prevalent form of religion. Since the birth of humanity populations have derived from nature aesthetic or spiritual sustenance and used it for creative ends [9]. Plants have a special role in religious and social ceremonies of every rural society [10]. Various religious and supernatural beliefs and folklores help in the prevention of destruction of plants. There are several examples of trees worship tradition in many parts of the world under all religions and beliefs. In the Muslim world, as well as in the middle east, sacred places are closely to the veneration of saints [11,12] and in many instances, sacred trees are connected with sacred graves/shrines and share the same supernatural powers to grant divine blessings to cure and to punish the offenders against the saint to whom the tree is dedicated and who endows them with their miraculous power [13-15]. The objective behind plant worship or plants used in religious festivals has always been their conservation and utilization in the most sustainable manner [7,16]. Plant worship as part of nature worship is generally believed to have begun in the initial stage of human society. However its origin and evolution are still an unfathomable enigma [17]. Many ethnic people have their tradition to worship different trees in different occasion. On the way if they come across the sacred tree they stop and tie a thread around the trunk of the tree or put flags near the tree [18]. Many religious plants where the culture and belief of the communities imbibed are seriously under threat and an urgent attention is therefore needed to preserve these plants.

**Figure 1:**
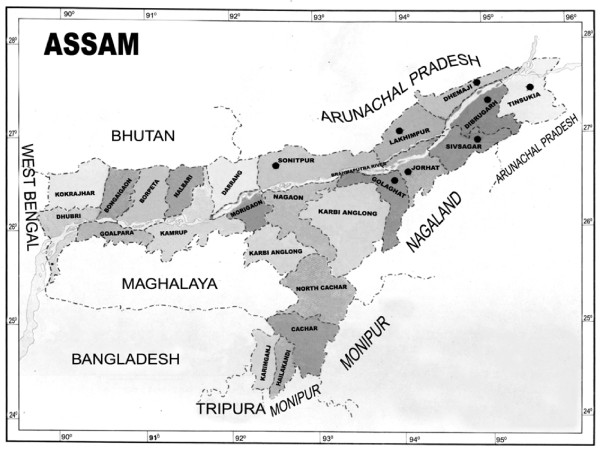
**Map of Assam. The black spots showing analyzed localities - Sonitpur, Lakhimpur, Dhemaji, Dibrugarh, Tinsukia, Sibsagar, Jorhat, Golaghat**.

Assam is situated in the North eastern region of India lying between 24°- 28° N and 90°- 96° E (Figure 1). The entire North Eastern region comprises 8 states (Assam, Arunachal Pradesh, Manipur, Meghalaya, Nagaland, Tripura, Mizoram and Sikkim). N.E. India with its rich floristic diversity is also inhabited by the largest number of tribes and they lead an intricate life totally dependent on the forest plants. Overall tribal population of this region accounts for more than 57 percent of the total population. In Assam alone the percentage of tribal population is 12.83. More than 25 communities, mostly tribal and mainly depending on plant resources for their day to day life inhabit in the different parts of the state. They have close association with and good knowledge about plant resources of their surroundings which form an integral part of their material and spiritual cultures. Assam has enormous ethno botanical wealth hand in hand with a rich cultural heritage but work on such aspects is very rare. From the different literatures so far published from this region it is known that there is a very little work done on the tribal people of Assam. However some experts [19-26] have few works on different problems of some tribal people of Assam. There is no specific work done so far on the plants used by the tribal people of Assam in different religious and cultural practices. It is in this background that the present study has been undertaken, which is aimed at the documentation of the plants related with religious and cultural practices in the Dobur Uie ritual of Mising people of Assam and their conservational practices.

**Figure 2:**
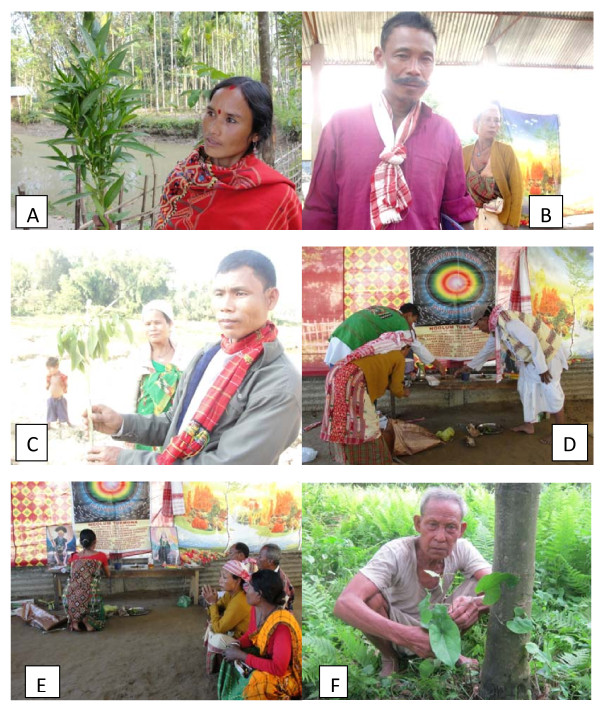
**Mising healers giving demonstration about the medicinal herbs and praying Dony-Polo**.

The Misings are one of the tribal communities of Assam. By faith they are the worshippers of 'Dony'(The Sun) and 'Polo' (The Moon) (Figure 2). They claim themselves as the sons of nature [23,27,28]. They perform number of religious practices in their life. The practices are comprised of various rites and rituals with prayer, offerings and sacrifices. They believe that after death of human beings their spirits which they call 'Uie' roam invisibly around them and these Uies are always hostile to human beings. There are several types of 'Uies' like Dobur Uie, Urom Uie, Taleng Uie, Gumin Uie etc and each type of 'Uies' is believed to cause particular type of problem. Dobur Uie causes all natural calamities like flood, erosion, drought, death etc. and therefore Dobur Uie is observed for getting rid of all these calamities. The types of ritual and offering are determined according to the nature of the spirits or 'Uie'. Generally the spirits causing the troubles are diagnosed by the 'Mibu' (Mising priest). To pacify the spirits the Misings perform different rituals by offering drinks (Apong- a kind of rice beer) and animals like chickens and pigs. Apong is inseparably associated with Mising culture. Without Apong no any ritual can be observed. In Dobur Uie it is sprinkled over the altar and also to the performers like the Mibus and other elderly persons sitting around the altar of the Dobur Uie to purify them. Apong is prepared in every household. About 10 - 15 plants are used in the preparation of Apong now a days. This number varies from place to place depending upon the availability of the plants. All plants used in Apong preparation have certain medicinal properties. Previously this number was 50 but it was alarmingly reduced to less than half number which is becoming a serious concern now a days. No any research work has been done on this aspect in this region. There are many plants used by the Mising people in different rituals. In this paper an attempt has been made to discuss the plants used in Dobur Uie. Many plants used by them in ritual purposes have medicinal properties. Studies on such plants used for worshipping gods and goddesses are carried out by different workers [6,29-36]

**Figure 3:**
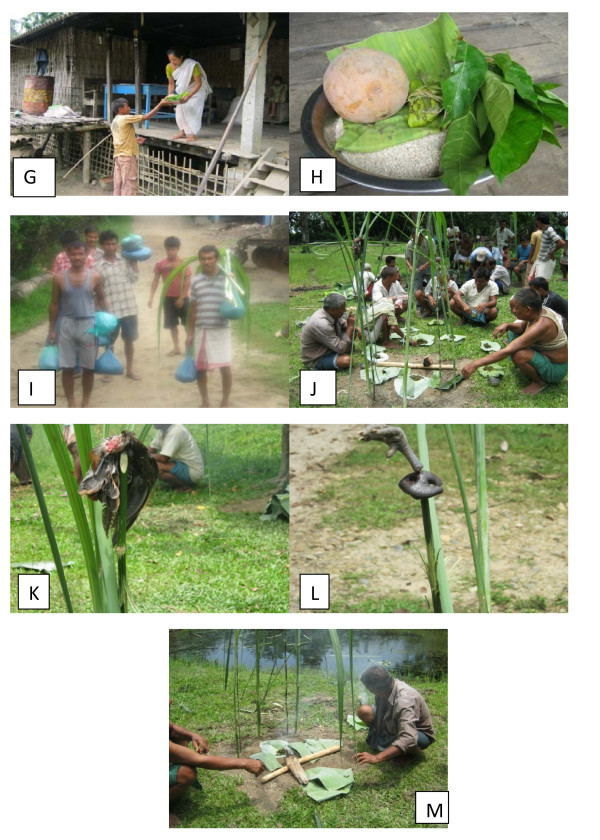
**Collection of 'Ajeng Dues' for performing 'Dobur Uie' ritual**.

Dobur Uie is observed in the month of May. People believe that the general welfare and prosperity of a village community depend on the blessings of Dobur Uie. It is observed at the outskirts of the village concerned so as to keep away the malevolent spirits from the village. A day is fixed (Generally Wednesday) for Dobur ritual considering all relevant aspects and convenience. In early morning of the 'Dobur day' some selected male folks proceed to the main entry points of the village. There they erect some structure specially designed braided leaves of 'Piro' plant (*Phragmites karka*), a kind of wild reed, its stems and bamboo to indicate prohibition against the entry of the outsiders into the village. After the completion of the prohibitory indicators at the entry points they come back to the village and they form a group consisting of 20 members approximately and start visiting the house holds of the village. Usually the visit starts from the eastern corner of the village. The members of the visiting group carry rods, sticks etc.in their hands and enter the courtyard of the houses. On entering they shout "Ajenge! Ajenge!Bilangka" (pay your fine and penalty) and at the same time they strike the platforms, walls etc of the house with rods and sticks. On hearing this the house wife comes out with food materials (Ajenge dues) (Figure 3) like rice, Apong, wild vegetables, chicken, pork etc and hand over them and after that the group move to the next households. In this way they visit every household of the village. They believe that beating the platforms or walls of the house will drive away the evil spirits from that house and eventually from the village They carry the collected materials to the bank of the river or stream. The ritual is performed by the Mibu (Mising priest). A temporary altar is prepared on the raised ground with specially designed sticks of Piro grass and bamboo and many other sacred plant leaves. First prayer is offered to their deities 'Dony - Polo'. Two symbolic idols simulating a snake swallowing an egg are made from 'Ruktak' plant (*Thelypteris angustifolia*) - a type of wild fern, a 'Tabong' (*Imperata cylindrica*) - a sharp grass and a split bamboo are placed on the altar facing the rising sun. Then the sacrifices of the animals like pigs and fowls are done. The heads, wings, legs of the poultries are mounted on specially designed sticks and erected them on the side of altar (Figure 3). Rice, Apong, and other collected eatables are served among the members taking part in the ritual.

## Methods

Areas analyzed: Research was carried out in these eight districts - Sonitpur, Lakhimpur, Dhemaji, Dibrugarh, Tinsukia, Sibsagar, Jorhat and Golaghat. These areas of Assam rich with traditional knowledge and a good vegetation cover were selected based on mopping up surveys during 2009-2010. The potential Mising areas were selected based on whether they still practice own social customs, beliefs, religious rites, taboos, totems, food habits, medicinal and agricultural practices, as it was observed that the utilization and conservation practices of the Mising tribes are intertwined with these. Also this kind of life style has created a proper understanding between them and nature, which has resulted in enormous amount of knowledge available with them. Exploration trips were conducted periodically and data on religious and conservation practices revealing information about their informal innovation were recorded with details. Initially, the stay in Mising areas was shorter periods, as the initial trips were aimed at establishing rapport with the Mising people. They will not pass the information to others so easily unless a cordial and social relationship with them is established. After making several trips to these areas good rapport could be established with them. Frequent field surveys were made. Headmen and Mibus of the hamlet were consulted to have easy access to the people and also to get uninhibited flow of information. Mising healers both men and women were invited for interview. They demonstrated about the different use of the medicinal plants (Figure 2). The Mising women are said to have better knowledge about the medicinal use of plants than men. Though they are not allowed to participate in Dobur Uie but due to their vast knowledge about the different uses of plants the women were interviewed after the end of the ritual. More over the women are very expert in identifying the plants used in Apong preparation. The women of different areas are interviewed to find out plant species used by them in the Apong preparation process.

For the best convenience field surveys were done in three phases. In the first phase information regarding their superstitious and religious beliefs, cultural practices, plants used in the Dobur Uie, source of collection of information and their names and address, medicinal use of the plants, description, local names and parts used in the magical treatment of the diseases are gathered. The practices of conservation were also noted.

In the second phase the informants were taken to the fields for identification of the plants in the field condition for collecting information on plant species they use and conservation practices they follow and also sustainable collection of the important plants is made. All necessary information about the plants and their status are gathered. Frequent visits to the Mising villages help in establishing social relationship with the tribal that help in gathering information on conservation and sustainable use of plant genetic resources. The information collected was compared with published literature and recorded following standard guide lines [37].

In the third phase the collected plant materials were made into herbarium specimens using the wet method [38] and deposited in Dhemaji College Herbarium (DCH) as voucher specimen. Some species are transplanted in Dhemaji College Botanical garden and inside the agro shed.

## Presentation of data

Systematic enumeration of the plants is arranged alphabetically. The families, Vernacular names, description of the voucher specimen, use of the plants in religious practice etc. are mentioned.

## Local status

The word Local status mentioned in the text is based on the availability of the plants used in Dobur Uie in those local areas where the research work has been carried out. This status may not be applicable in other places. The Mising people have themselves categorized the plants as available, rare and very rare.

## Abbreviations used

(Eng): English; (Ass): Assamese; (Mis): Mising; (Nep): Nepali; (San): Sanskrit; (Hin): Hindi; (Ben): Bengali.

## Results

***Acorus calamus ***L.(**Acoraceae**)

**Vernacular names**: Sweet flag (Eng), Bos(Ass); Alokoni (Mis); Bojho (Nep.) Bach: (Ben)Vacha (San)

DCH 5063

**Description**: A marshy, rhizomatous perennial herb.

**Parts used**: Rhizome

**Religious virtue**: People believe that spirits, devils cannot enter due to its odour. They put the rhizome at the four corners of the altar or at he entrance of the ritual site.

**Medicinal use**: It cures bronchitis, rheumatic pain, diarrhoea, flatulence, Pneumonia and cough.

**Local status**: Rare

***Ageratum conyzoides ***L. **(Asteraceae)**

**Vernacular names**: Eng: Goat weed; San: Visamustih; Hin: Visadodi; Ben: Uchunti; Ass, Mis: Gendelabon; Nep: Ilame jhar; Man: Khonjainapi DCH 5001

**Description**: A hairy annual weed, 5 m-1 m tall; leaves opposite, hairy on both sides; margin toothed; flowers pale in heads.

**Parts used**: The flowers, leaves, roots

**Religious virtue**: Flowers are used in the preparation of Apong

**Medicinal use**: Infusion of roots is used as appetizer and ophthalmic; leaves are used to stop bleeding

**Local status**: Available

***Alpinia allughas ***(Retz.) Rosc. (**Zingiberaceae)**

**Vernacular names**: Ass, Mis: Tora DCH 5006

**Description**: Rhizomes tuberous, perennial, Leafy stem 1-2 meter high

**Parts used**: Leaf and rhizome

**Religious virtue**: The leaves of the plant are used as food wrappers or dishes in every Mising religious festival. The people regard the plant as sacred one.

**Medicinal use **: Rhizomes are used in gout and colic

**Local status**: Rare

***Alpinia malaccensis ***Rosc. (**Zingiberaceae**)

**Vernacular names**.: Ass: Kaupat, Mis: Lisin DCH 5010

**Description**: Rhizomes tuberous, perennial, Stems 1.5-3 meter high

**Parts used**: Leaf and rhizome

**Religious virtue**: Same as in *A.allughas*

**Medicinal use **:Rhizomes are used for treatment of sores.

**Local status**: Rare

***Ananas comosus ***(L.) Merr. **(Bromeliaceae)**

**Vernacular names**: Eng: Pineapple; San: Anamnasam; Hin: Ananas; Ben: Anaras; Ass: Matikathal; Nep: Bhui Katahar; Man: Keehom; Kar: Parokjongphong; Ngl: Yeangkong Peyong. Mis: Anaras DCH 5311.

**Description**: A herbaceous perennial plant; leaves many, spirally arranged, linear-lanceolate, toothed on margins; inflorescence small, reddish, terminal, ovoid.

**Parts used**:Tender leaf

**Religious virtue**: Tender leaf base is used in Apong preparation

**Medicinal use**: Leaf base is crushed and the extract is given one time daily for amoebic dysentery and intestinal worms

**Local status**: Available

***Artocarpus heterophyllus ***Lamk. **(Moraceae)**

**Vernacular names **: Eng : Jack fruit; San : Panasah; Hin : Kathal; Ass, Mis : Kathal; Ben : Kathal; Nep : Katahar; Man : Theibong. DCH 6331

**Description **: An evergreen tree, 18-25 m in height; leaves simple, alternate, coriaceous, entire, shiny; male flowers crowded on cylindrical receptacles; female flowers crowded on globose receptacles; fruits fleshy, many, oblong or round, covered with tubercles.

**Parts used **: Roots, seeds.

**Religious virtue**: Matured leaves are used in Apong preparation. Leaves are also used as dish in religious festivals.

**Medicinal use**: Roots are used for diarrhea

**Local status**: Available

***Asparagus racemosus ***Willd (**Liliaceae**)

**Vernacular names**:Eng: Wild Asparagus; San: Shatavari: Hin: Satawari;Nep: Kurilo, Mis: Otmul;Ass: Satmul; Ben: Satamuli; Man: Nunggarei. DCH 6019

**Description**: A climbing slender plant with curved spines; leaves reduced to scales, leaf like cladodes succulent and green, flowers white in simple recemes; fruits three lobed, mature fruits are red in colour.

**Parts used**: Tuberous roots, whole plant

**Religious virtue**: In Lakhimpur district many people use whole plant near the entry of Dobur Uie venue. They believe that this plant will guard the people from the bad spirits.

In Dhemaji district the tuberous roots are used in the preparation of Apong.

**Medicinal use**: Root decoction is used as health tonic, it is diuretic, ophthalmic, galactagogue, aphrodisiac and carminative.

**Local status**: Very rare

***Bambusa tulda ***Roxb. **(Poaceae)**

**Vernacular names **Jati banh (Ass); Peka (Hin); Taru Bans (Nep); Jati dibang (Mis)

DCH 5054

**Description**: Tufted bamboo reaching 30-50 meters in height.

**Parts used**: Root, stem. leaves

**Religious virtue**: Bamboo sticks and leaves are used in the main entrance of the Dobur Uie and in the four corners of the altar. The head and the feathers of the sacrificed chickens are hung on the bamboo sticks near the altar.

**Medicinal use **: The decoction of roots taken internally to promote flow of urine.

**Local status**: Available

***Centella asiatica ***(L.) Urban **(Apiaceae)**

(Syn. *Hydrocotyle asiatica *L.)

**Vernacular names**: Eng: Indian Pennywort; San: Brahmi, Mandukaparni, Hin: Brahamamanduki; Ben: Tholkuri, Ass, Mis: Manimuni; Nep: Ghod tapre. DCH 6007

**Description**: A creeping herb with rooting nodes and long internodes; leaves reniform, toothed, flowers in clusters, pink.

**Parts use**: Whole plant.

**Religious virtue**: The plant is used in the preparation of Apong. It is given in Ajenge Dues. Mising peope believe that if they consume the plant in the Dobur Uie day as vegetable or as raw they cannot be disturbed by any kind of spirits.

**Medicinal use**: The leaves are used in amoebic dysentery or any kind of liver problem.

**Local status**: Available

***Cinnamomum tamala ***Nees & Eberm. **(Lauraceae)**

**Vernacular Names**: Eng: Indian cassia lignea; San: Tamala patra; Hin: Tezpat; Ben: Tezpat; Ass, Mis: Tezpat; Nep: Tezpat; Man: Tezpat; Kar: Tezpat; Miz: Hnahrimtui DCH 6103

**Description**: A small evergreen tree 4.5 m in height; leaves simple, opposite, lanceolate, glabrous, entire; flowers pale yellowish, in axillary panicle.

**Parts used**: Leaves.

**Religious virtue**: Leaves are used in Apong preparation and are offered in Ajenge Dues

**Medicinal use**: The leaves are useful in gonorrhoea, rheumatism, diarrhea, enlargement of spleen and diabetes.

**Local status**: Rare

***Clerodendrum colebrookianum ***Walp. (**Verbenaceae)**

**Vernacular names**. Pakkom (Mis); Nephaphu (Ass) DCH 5019

**Description**: Shrub with foetid smell; 1.5-2 meters high, Leaves ovate, Flowers white.

**Parts used**: Leaves

**Religious virtue**: The leaves of the 'Pakkom'are included in the Ajenge Dues

**Medicinal use**: The leaves are used to kill the intestinal worms. Tender leaves are boiled and the soup is used for reducing blood pressure.

**Local status**: Available

***Dillenia indica ***L. (**Dillenniaceae**)

**Vernacular names**. Elephant apple (Eng), Outenga (Ass): Sompa: (Mis), Nep: Chalta, Bhavya: (San) DCH 5013

**Description**: A big tree; leave 10-15 cm broad, Flowers large, white, the fruits are used as vegetables

**Parts used**: Sepal of the fruit.

**Religious virtue**: The fruits are included in 'Ajenge Dues'. The sepals are sometimes used as 'Diya'or oil lamp (Oil lamp : A sepal containing wick dipped in mustard oil)

**Medicinal use**: Fleshy calyx is used for stomach disorder. The jelly like pulp of the fruit is applied to scalp for curing dandruff and falling hairs.

**Local status**: Available

***Ficus racemosa ***L. (**Moraceae**)

**Vernacular names**.: Eng:Cluster fig; Mis: Tajjig; San: Udumbarah); Ass: Jagnya dimaru); Nep: Dumri, Hin: Gular; Ben:Dumur DCH 5057

**Description**: A middle sized deciduous tree, fruits are in large cluster on short leafless branches, leaves elliptic, ovate or ovate lanceolate.

**Parts used**: Leaves, latex

**Religious virtue**: The tender leaves are given in 'Ajenge Dues'. In all religious festivals of Mising people the leaves are used as the primary curry item.

**Medicinal use**: The latex is used for piles and diarrhoea; Powdered dry leaves are mixed with honey and given in bilious affections.

**Local status**: Available

***Gomphostemma parviflora ***Wall. **(Lamiaceae)**

**Vernacular names**: Ass, Mis: Bhedaitita DCH 6434

**Description**: A stout undershrub with quadrangular stem covered with glandular hairs; leaves simple, opposite, short petioled; flowers zygomorphic, in axillary, double row of cymes, the pairs forming false whorls and are in sessile clusters round the stem, flowers fade yellow.

**Parts used**: Leaves.

**Religious virtue**: The tender leaves are used in Apong preparation

**Medicinal use**: Leaves are used in Malaria.

**Local status**: Very rare

***Imperata cylindrica ***(L.) Raeusch. (**Poaceae**)

**Vernacular names**: Eng: Thatch grass, San: Darbha, Mis: Kase, Tabong: Ass: Ulu kher: Nep: Siru: Hin, Ben: Ulu DCH 6336

**Description**: Erect perennial grass, 1-2 meter in height. Leaf blades are very sharp

**Parts used**: Leaf and root

**Religious virtue**: The leaves are tied with fronds of *Thelypteris multilineata *at the south corner of the altar made for Dobur Uie. It symbolizes that no any other spirits can come and disturb people of the village.

**Medicinal use **: Root is used for wounds and piles. It is anthelmintic. Decoction of root is taken in diarrhea and dysentery.

**Local status**: Available

***Kaempferia rotunda ***L. **(Zingiberaceae)**

**Vernacular names **: Eng: Indian crocus; San: Bhumi champaka; Hin: Bhuichampa; Ben: Bhui champa; Ass: Bhumi champa; Nep: Bhuichampa; Man: Yai-Thamna-manbi; Kar: Michove; Miz: Tuktinpar DCH 5999

**Description**: A small, erect plant with perennial rootstock and very short stem; leaves simple, erect, oblong or ovate-lanecolate, acuminate, variegated green above, tinged with purple below; flowers in crowded spikes fragrant, white, lip purple.

**Parts used**: Tubers

**Religious virtue**: Tuber of the plant is used in Apong preparation. The Mising of Lakhimpur district believes that growing the plant brings peace in the family.

**Medicinal use**: The tubers are used for wounds, ulcers, tumours, swellings and gastroenteritis.

**Local status**: Very rare

***Leucas plukenetii ***(Roth) Spreng. **(Lamiaceae)**

**Vernacular names**: Eng: Thumbe; San: Dronapuspi; Hin: Chota halkusa; Ben: Sada halkusa; Ass, Mis: Boga Doron; Nep: Dronapuspa; Man: Mayanglambum. DCH 6312

**Description**: Small hairy, square stemmed, annual herb found in open fields and waste lands. The leavesof the plants are ovate-lanceolate, lobed, thin and hairy with a tapering base; flowers white, small, in dense terminal clusters, sessile.

**Parts used**: Leaves.

**Religious virtue**: The leaves are used in Apong preparation. It is not used by all Mising

**Medicinal use**: The leaves are used as vegetables. Leaf juice is used in sinusitis

**Local status**: Available

***Microsorum punctatum ***(L.) Copel (**Polypodiaceae**)

**Vernacular names**: Eng: Climbing bird's nest fern, Ass: Kapau dhekia; Mis: Ising Okang DCH 6216

**Description**: Terrestrial or epiphytic, Fronds sre sessile, leaf blades simple, reticulate venation, sori are irregularly scattered on the abaxial surface.

**Parts used**: Leaves

**Religious virtue**: Leaves are used in Apong preparation in Dhemaji and Lakhimpur district.

**Medicinal use**: Leaf juice used as purgative, diuretic and healing wounds.

**Local status**: Rare

***Musa paradisiaca ***L. **(Musaceae)**

**Vernacular names**. Ass: Kach kol; Nep: Kera, Hin: Kela; San: Kadali; Mis: Kopak. DCH 5091

**Description**: Stoloniferous plant; leaves with sheathed petiole up to 9 meter long, flowers in a terminal spike.

**Parts used**: Leaves, fruits

**Religious virtue**: Leaves and leaf sheaths are used as plates or dishes in the ritual. The materials required for observing Dobur Uie are kept in the leaf or leaf sheath dishes.

**Medicinal use**: Fruits are used for chronic dysentery.

**Local status**: Available

***Naravelia zeylanica ***(L.) DC. **(Ranunculaceae)**

**Vernacular names**: San: Dhanavalli; Ben: Chagalbati; Ass: Goropchoi, Nep: Ras gagri; Meg: Jyrmailasam, Behalisham (Garo) DCH 6636

**Description**: A woody stout climber; leaves opposite, 2-foliate, terminal leaflet ending in tendril, leaflets ovate-lanceolate;flowers yellow, in axillary and terminal panicles.

**Parts used**: Leaf.

**Religious virtue**: Leaves are used in Apong preparation. The Mising of Dhemaji believe that if they grow this plant inside their compound the bad spirits can not disturb them.

**Medicinal use**: The leaves are anthelmintic; they are useful for wounds and ulcers.

**Local status**: Very rare

***Oldenlandia corymbosa ***L. (**Rubiaceae**)

**Vernacular names**: Eng: Diamond flower; San: parapatah; Hin: Daman Pappar;

Ass: Bonjaluk DCH 6212

**Description**: A much spreading, annual herb, erect or spreading; leaves simple, opposite, sessile, flowers white, pedicelled, solitary.

**Parts used**: whole plant

**Religious virtue**: It is commonly used in Apong preparation. In Sibsagar district it is given in Ajenge Dues.

**Medicinal use**: The plant is diuretic, stomachic, carminative and used as liver tonic. It is also used in jaundice.

**Local status**: Available

***Oryza sativa ***L. **(Poaceae)**

**Vernacular names**: Eng: Rice; Ass:Dhan; Mis: Aam. DCH 6001

**Description**: Annual cereal, Stem erect, cylindrical, hollow except in nodes, leaf blade long, lanceolate, acuminate, inflorescence is partly covered by leaf sheath, Spikelets are borne either singly or in cluster, flowers bracteates.

**Parts used **: Seed, Straw

**Religious virtue**: Rice is indispensable item in Apong preparation. Straw is burnt and used in 'Chhai Mod' (Ash liquor). The colour of commonly used Apong is white but the colour of 'Chhai Mod' is smoky in colour. Chhai Mod is prepared only in the special occasion or any religious festivals. Important guests are entertained by offering 'Chhai Mod'. The straws are also used to filter the Apong.

**Medicinal use**: Rice-wash water (water used to wash rice before cooking) is used in diarrhea and dysentery

**Local status**: Available

***Phragmites karka ***(Retz.) Trin.ex Steud. (**Poaceae**)

**Vernacular names**.: Eng: Wild reed; Mis:Piro; Ass: Nal Khagari; Nep:Narkat; Ben: Khagra DCH 5066

**Description**: Perennial, stem cylindrical, 2-3 meter high, leaves alternate, lanceolate.

**Parts used**: whole plant, root

**Religious virtue**: The plant is mostly used by the Mising community in religious festivals like Dobur Uie and Ali Ai Ligang. In Dobur Uie four 'Piro' plants are transplanted at the four corners of the Dobur Uie altar' Mising regard this plant as the most sacred plant.

**Medicinal use**: Roots are cooling, diuretic and very useful in Diabetes.

**Local status**: Rare

***Psidium guajava ***L. **(Myrtaceae)**

**Vernacular names**: Eng: Guava tree; San: perala, Perukah; Hin: Amrud; Ben:Peyara; Ass: Madhuriam; Nep: Ambak; Man:Pungdol. DCH 6010

**Description**: A small tree; leaves simple, opposite, elliptic-oblong, glabrous above; flowers white, fragrant, in axillary cymes.

**Parts used**: Tender leaves.

**Religious virtue**: It is very commonly used by the Mising in Apong preparation.

**Medicinal use**: Tender leaves are used in Amoebic dysentery

**Local status**: Available

***Pueraria tuberose ***(Roxb.ex Willd.)DC (**Papilionaceae**)

**Vernacular names**: Eng: Indian Kudzu; San: Vidari, Bhumi Kusmandah; Ass: Bhuin Komora; DCH 6455

**Description**: A large, herbaceous twiner with very large tuberous roots; leaves 3 foliolate, leaflets broadly ovate; flowers blue or purplish blue, in raceme; fruits membranous, flat, jointed clothed with long, silky, bristly brown hairs.

**Parts used**: Tuberous roots.

**Religious virtue**: Tuberous roots are used in Apong preparation

**Medicinal use**: Tubers are used for fever.

**Local status**: Very rare

***Sarcochlamys pulcherrima ***(Roxb.) Gaud. (**Urticaceae**)

**Vernacular names**. Eng. Duggal fibre tree; Mis :Ombe; Ass: Mesaki DCH 5101

**Description**:A small evergreen branched tree or large shrub upto a height 5 meter, Stem brownish, rough and warty, leaves in dense clusters at the apex of the branches, 12-20 cm long, dorsal surface shining green and ventral surface white.

**Parts used**: Leaves

**Religious virtue**: Mising people consider the plant as sacred plant. They make special food item from the tender leaves of the plant in any religious festivals, given in 'Ajeng Dues'.

**Medicinal use**: Leaves are useful for diarrhea and dysentery, they are carminative and digestive.

**Local status**: Rare

***Scoparia dulcis ***L. **(Scrophulariaceae)**

**Vernacular names**: Eng: Sweet broomweed; Ass : Seni bon; Man : Maipuipin.

DCH 6135

**Description**: A much branched, herbaceous plant of about 70 cm height; leaves opposite, lanceolate, dentate, flower four lobed, white with staminal hair, fruit a dentate margined small globular capsule with free central placentation.

**Parts used **: Leaves.

**Religious virtue**: Leaves are used in Apong preparation

**Medicinal use**: Leaves are used for fever, cough and diabetes.

**Local status**: Available

***Spilanthes paniculata ***Wall.ex DC (**Asteraceae**)

**Vernacular names**. Eng: Brazil cress, Toothache plant; Mis: Marsang; Ass: Jati malkathi DCH 5045

**Description**: Herb upto 40 cm. in heght; leaves opposite, dentate, petiolate, elliptic-lanceolate; flowers in capitulum, yellow.

**Parts used**: Whole plant, flower

**Religious virtue**: The plant is very popular among the Mising community; special food item is prepared from this plant in religious festival. The poor people offered this plant along with the 'Ajeng Dues' in Dobur Uie

**Medicinal use**: The inflorescence relives toothache, bronchial trouble and ulcers inside the mouth, it has strong local anaesthetization and also used for dysentery.

**Local status**: Available

***Thelypteris angustifolia ***(Willd.)Proctor (**Thelypteridaceae**)

**Vernacular names**: Mis: Ruktak; Nep: Koche DCH 5111

**Description**:Terrestral fern, Fronds large, pinnate, sessile, found in marshy place.

**Parts used**: Whole plant, rhizome

**Religious virtue**: The entire plant is placed ine corneer of the altar. The tip of the plant and tip of the leaves of 'Piro' are tied together near the altar of the Dobur Uie

**Medicinal use**: Juice of the rhizome about four teaspoons three times a day given for indigestion or any stomach problem.

**Local status**: Rare

***Zanthoxylum nitidum ***(Roxb.) DC (**Rutaceae**)

**Vernacular names**: Eng: Toothache tree; San:Tumburuh; Hin: Tezbal; Ass: Tezmooi DCH 6020

**Description**: A large, scandent, ever green shrub with prickles, shrubs or woody climbers, leaves compound, flowers yellow, fruits reddish, subglobose.

**Parts used**: Leaf, stem bark

**Religious virtue**: Leaves are used in Apong preparation

**Medicinal use**: Stem bark is used for toothache or any gum problem, it is carminative and stomachic.

**Local status**: Very rare

### Plant Conservation Practices of Mising

Mising people have a rich tradition of religion and medicinal practices. They collect the plants from wild state and conserve them around their residences. They have good knowledge about the status of the plants. This knowledge has been acquired by their long experiences. They have been using the plants for different purposes since time immemorial. So they have good knowledge about the use and availability of the plants. Here the 'availability' means the 'status'of the plants. Some plants like *Pueraria tuberose, Zanthoxylum nitidum, Naravelia zeylanica, Gomphostemma parviflora, Asparagus racemosus, Kaempferia rotunda *are not found easily in those areas where the research work has been carried out. Previously these plants were said to be found abundant but now they are in 'very rare' condition. In this way they categorized the plants in different status. They can easily identify the plants which are locally threatened or abundant, rare or very rare depending on the availability of the plants and accordingly they take special care to conserve such threatened species in their garden. So there is an urgent need of conservation of their rich traditional knowledge before their extinction. The old village heads or elderly persons of the research areas were interviewed. They expressed about their constrains for leaking out the information about the medicinal plants at the beginning but later on we somehow could motivate them and collected few information. They strongly believe that if they leak the information the efficacy of the drugs will be lost. If this trend continues their rich traditional knowledge will be lost along with their death. So the need of conservation of their knowledge is very essential. The status which they mentioned may not be applicable in other places. It is strictly confined to their areas only and therefore the word 'Local status' is mentioned. They collected many important plants from the forest and transplanted in the gardens. The authors personally visited some of their gardens and found some important plants like *Pueraria tuberose, Zanthoxylum nitidum, Naravelia zeylanica, Gomphostemma parviflora, Asparagus racemosus, Kaempferia rotunda *etc.

## Discussion

In the Dobur Uie day the Mising people will take wild vegetables with their meal. In the previous day of the ritual the Mising women go to the forest for collecting wild vegetables for the next day. 20-30 varieties of wild vegetables were collected in the earlier time but this number comes down to 10-15 at present. On the other hand the alarming decrease of Apong plants from 50 to 10-15 is becoming a serious concern. As stated by the informants many plants got eliminated from the locality may be due to wanton felling of the trees or may be indiscriminate collection of the plants. Moreover other tribal people except the Mising also use the same plants for Apong preparation resulting over exploitation of these plants. The informants stated that if this trend of decreasing the number of plants used in different ritual practices continues then the situation of cultural threat may arise one day or other. This situation is found in all Mising populated areas undertaken for this study. In this paper altogether 30 plants under 23 families were discussed. Plants used for wild vegetables, Apong preparation and for Dobur Uie celebration are found in wild state and many of them like *Zanthoxylum nitidum*, *Asparagus racemosus, Sarcochlamys pulcherrima, Pueraria tuberose, Phragmites karka, Naravelia zeylanica, Gomphostemma parviflora, Ficus racemosa, Dillenia indica, Clerodendrum colebrookianum, Cinnamomum tamala, Acorus calamus, Alpinia malaccensis, Alpinia allughas *have been domesticated. They sometimes sell the vegetable parts in the local market for their livelihood. These benefits coming out from the market will typically go to improving living standards of their families. This makes a clear link between conservation and development. Communities can contribute to biodiversityby collecting and propagating indigenous seeds, planting and tending both indigenous and plantation trees in their forests and their private farms and policing against illegal harvesting of animals and tree products. These plants seem to be threatened to get eliminated in their natural habitats due to the over exploitation. Because other tribal communities also collect the plants from the same source. So the domestication of these plants is a need for conservation. Keeping this in view the plants used in Dobur Uie, Apong preparation or vegetables are locally categorized in three divisions rare, very rare and available. Eight rare plants and six very rare plants have been identified to be used in Dobur Uie celebration. Very rare plants like *Zanthoxylum nitidum, Pueraria tuberose, Naravelia zeylanica, Gomphostemma parviflora, Asparagus racemosus*, *Kaempferia rotunda *and need urgent conservation for their sustainable use. The plants locally found and used in Dobur Uie in rare status are *Thelypteris angustifolia*, *Sarcochlamys pulcherrima, Phragmites karka*, *Microsorum punctatum*, *Cinnamomum tamala*, *Alpinia allughas*, *Alpinia malaccensis *and *Acorus calamus. *For different ritual purposes these religious plants are frequently used by the Mising people so the Mising people are trying their best to protect the plants in situ or ex situ condition from elimination. The rest 16 plants though are found locally available but due to lack of awareness of the people for sustainable use of the plants many such plants like *Spilanthes paniculata*, *Clerodendrum colebrookianum*, *Dillenia indica*, *Centella asiatica *though are found in available status but due to unsustainable use and over exploitation the number of these plants is decreasing gradually and therefore they need an urgent protection. As the Mising culture and plants have inseparable linkage so to protect the rich Mising culture the plants need to be conserved.

The strong motive behind observing Dobur Uie is the sense of fear or reverence shown by the Mising people towards god or deities. Be it a patch, a single or cluster of sacred trees a lot of conservation concern has been imbibed into the Mising communities. Irrespective of the motivating force behind conferring the sacred or religious status to a plant, it may be said that this process saves the plants from wanton felling. Through this project an attempt has also been made to make them aware of the status and sustainable use of the plants.

A number of folk medicinal claims used by Mising people in Dobur Uie are reported in this communication. Among these, some interesting and new claims relate to the treatment of different ailments of human beings such as diarrhea, dysentery, malaria, indigestion, gastroenteritis, high blood pressure, intestinal worms etc. About 34 diseases have been described for the treatment of different ailments. Diarrhoea, dysentery, indigestion, flatulence, stomach problems, liver problems are found very common among the diseases. Information on the effectiveness to cure the ailments by using these species is based on continuous use by succeeding generations of Mising people. However these claims need to be further tested using standard scientific methodologies. Local names are given in different regional languages for the easy identification of the species.

## Conclusion

In the present study 30 plants belonging to 23 families have been identified as medicinal plants used by the Mising people of Assam in Dobur Uie ritual. These plants are used in the treatment of some very common ailments like diarrhea, dysentery, indigestion, flatulence, stomach problems, liver problems etc. All these diseases are water born and this can be explained that the water taken from ponds or from other free sources in rural areas are not hygienic and so the children are easily infected by these diseases. The Mising people believe that all big trees are abodes of gods and deities and therefore they are sacred and should not be harmed, they try to save these plants by all means This perception of nature plays a positive role on the protection of the vegetation around their dwelling places. That is why many scholars advocate that people living in modern society learn from the minority people to respect the environment rather than depredating it [6-8,14,20,21] Mising people adore Apong, it is used daily and in every ceremony, no any festival takes place without the use of Apong. Apong is offered to the guests to show respect. In Dobur Uie Apong is used from beginning to the end of the festival. But the reducing trend of Apong plants in the forest is now becoming a serious concern for the Mising community as their cultural identity is intertwined with these plants. Domestication of religious and wild vegetable plants is a good sign for conservation point of view. Every Mising family grows some wild vegetable plants like *Gomphostemma parviflora, Clerodendrum colebrookianum, Ficus racemosa*, *Sarcochlamys pulcherrima *etc. in their gardens for consumption and sale. These plants can help overcome the deficiency of nutritional constituents, especially in rural areas. It is important to promote consciousness about the food habits and accept wild food plants like the cultivated ones. Thus they become conscious about conserving their surrounding plant resources. Though the use of plants in Dobur Uie varies from place to place but some plants like *Alpinia allughas*, *Alpinia malaccensis*, *Imperata cylindrica, Thelypteris angustifolia*, *Phragmites karka *are found common to all places. We suggest that the traditional knowledge of the Mising people could provide useful information in finding new drugs that contribute to human welfare. So the most urgent need is to rescue and record the traditional knowledge on plants in the form of digitized database before its extinction.

## Consent

Permission was granted by the Mising people shown in figures 2 and 3. They have declared that they have no objection to the publication of their pictures in the journal. These pictures have not been published previously.

## Competing interests

The authors declare that they have no competing interests.

## Authors' contributions

US conducted field survey, interviews with the healers, village heads, head of the religious institutions, elderly men and women. Herbarium specimens were identified and finalized with SP. SP helped to take the photographs and to collect and analyze data. Both the authors examined the manuscripts minutely and approved as final manuscript

## References

[B1] Kumbhojkar MS Upadhyaya AS Kulkarni DK Jain SK Religious Forest Patches among Mahadeo Koli Tribal localities - Social, Cultural and Environmental Relationship Ethnobiology in Human Welfare 1996 New Delhi; Deep publications 349 351

[B2] Archana Godbole Jain SK Role of Tribals in Preservation of Sacred Forests Ethnobiology in Human Welfare 1996 New Delhi; Deep publications 345 348

[B3] Rajendran SM Agarwal SC Medicinal plants conservation through sacred forests by ethnic tribals of Virudhunagar district, Tamil Nadu Indian Journal of Traditional Knowledge 2007 6 2 328 333

[B4] Hema Sane Vinaya Ghate Sacred conservation practices at species level through tree worship Ethnobotany 2006 18 46 52

[B5] Priyadarsan Sensarma The sacred and religious plants in Tantra sastra Ethnobotany 1995 7 51 61

[B6] Sinha RK Jain SK Conservation of cultural diversity of indigenous people essential for protection of biological diversity Ethnobiology in Human Welfare 1996 New Delhi; Deep publications 280 283

[B7] Ravishankar T Jain SK Role of indigenous people in the conservation of plant genetic resources Ethnobiology in Human Welfare 1996 New Delhi; Deep publications 310 314

[B8] Smith Eric Alden Conservation and subsistence in small scale societies Annual Review of Anthropology 2000 29 493 524 10.1146/annurev.anthro.29.1.493

[B9] Airzpe L Culture and environment Nature & Resources 1996 32 1 121638331

[B10] Manilal KS Linkages of ethnobotany with other sciences and discipline Ethnobotany 1989 1 15 24

[B11] Goldziher I Stern SM Muslim studies 1971 London: George Allen and Unwin Ltd

[B12] Westermarck E Ritual and Belief in Morocco 1968 New York University Books

[B13] Dafni A Why are rags tied to the sacred trees of the holy land? Econ Bot 2002 56 315 327 10.1663/0013-0001(2002)056[0315:WARTTT]2.0.CO;2

[B14] Dafni A The supernatural characters and powers of sacred trees in the Holy Land J Ethnobio Ethnomedicine 2007 3 10 10.1186/1746-4269-3-10 PMC1820775 17319970

[B15] Dafni A On the typology and the worship status of sacred trees with a special reference to the Middle East J Ethnobio Ethnomedicine 2006 2 26 41 10.1186/1746-4269-2-26 PMC1500805 16700917

[B16] Aizhong Liu Shengji Pei Chen Sanyang Plant Worship of the Yi People in Chuxiong of Yunnan, China Ethnobotany 1999 11 1 8

[B17] Zhang QG Chen LS Anthropology of Religeon 1993 Sichuan, Chengdu; Sichuan University Press

[B18] Dafni A Levy S Lev A The ethnobotany of Christ's Thorn Jujube (*Ziziphus spina christi*) in Israel J Ethnobio Ethnomedicine 2005 1 12 10.1186/1746-4269-1-12 PMC1277088 16270941

[B19] Borthakur SK a. Less known medicinal uses of plants among the tribes of Mikir Hills Bull. Bot. Surv. India 1976 18 1-4 166 171

[B20] Borthakur SK Sarma UK Jain SK Ethnoveterinary medicine with special reference to cattle prevalent among the Nepalis of Assam, India Ethnobiology in Human Welfare 1996 New Delhi; Deep publications 197 199

[B21] Borah S Das AK Boruah AM Borah J Ethnomedicinal plants used by the Mishing communities for analgesic and anti-inflammatory properties in India Ethnobotany 2009 21 66 69

[B22] Doley D Jawahar jyoti Kuli The socio economic life of the Miri tribe TheMisings: Their History and Culture 1998 Ayir Publication, Guwahati 91 104

[B23] Doley B The Misings, Sons of the Nature 1997 Guwahati, Assam

[B24] Hajra PK Boissya AK Jain SK Ethnobotanical notes on Miris (Misings) of Assam Plains Glimpses of Indian Ethnobotany 1981 Oxford & IBH publishing Co., New Delhi 161 167

[B25] Sharma UK Boissya CL Menstrual Problems : Ethnobotany Practices among the Mising tribes in Dhemaji district of Assam Ad Plant Sci 2003 16 1 17 21

[B26] Ghate VS Plants in patra puja. Notes on their identity and utilization Ethnobotany 1998 10 6 15

[B27] Nath D Jawahar jyoti Kuli The Mising Society in Transition The Misings: Their History and Culture 1998 Ayir Publication, Guwahati 132 136

[B28] Pegu N Jawahar jyoti Kuli The Misings: a colourful tribe of the Brahmaputra valley The Misings: Their History and Culture 1998 Ayir Publication, Guwahati 34 45

[B29] Dixit G Fire Sacrificial plants, Geobios New Reports 1997 16 8 47 48

[B30] Gadgil M Vartak VD The sacred grooves of western ghats in India Econ. Bot 1975 30 2 152 166

[B31] Ghate VS Plants in patra puja. Notes on their identity and utilization Ethnobotany 1998 10 6 15

[B32] Hajra PK Jain SK Plants in medico-religious beliefs in Sanskrit literature Manual of Ethnobotany 1987 Lucknow, India 117 124

[B33] Jain SK Dictionary of Indian Folk Medicine and Ethnobotany 1991 Deep publication New Delhi

[B34] Schultes R Hoffmann A Plants of Gods 1979 Mc Graw Hill Book Co., New York 1 192

[B35] Upadhyaya AS Kumbhojkar MS Kulkarni DK Ethno-medico botany of some sacred plants of western Maharashthra Ethnobotany 1997 9 65 68

[B36] Acharya Varahamihir Thakur S Brihat Samhita 1984 Pravin Pustak Bhandar, Rajkot

[B37] Rao RR Jain SK Methods and techniques in ethnobotanical study and research: some basic consideration Methods and approaches in Ethnobotany 1989 Society of Ethno-botanists, Lucknow 13 23

[B38] Fosberg FR Sachet MH Manual for Tropical Herbaria 1965 Netherlands

